# Quantitative Evaluation of Very Low Levels of HIV-1 Reverse Transcriptase by a Novel Highly Sensitive RT-qPCR Assay

**DOI:** 10.3390/life12081130

**Published:** 2022-07-27

**Authors:** Francesca Marino-Merlo, Valeria Stefanizzi, Agnese Ragno, Lucia Piredda, Sandro Grelli, Beatrice Macchi, Antonio Mastino

**Affiliations:** 1 Department of Chemical, Biological, Pharmaceutical, and Environmental Sciences, University of Messina, 98166 Messina, Italy; fmarino@unime.it (F.M.-M.); agnese.ragno96@gmail.com (A.R.); 2 Department of Chemical Science and Technology, University of Rome “Tor Vergata”, 00133 Rome, Italy; valeriastefanizzi1995@gmail.com (V.S.); macchi@med.uniroma2.it (B.M.); 3 PhD Course in Microbiology, Immunology, Infectious Diseases, and Transplants (MIMIT), University of Rome Tor Vergata, 00133 Rome, Italy; 4 Department of Biology, University of Rome “Tor Vergata”, 00133 Rome, Italy; lucia.piredda@uniroma2.it; 5 Department of Experimental Medicine, University of Rome “Tor Vergata”, 00133 Rome, Italy; grelli@med.uniroma2.it; 6 The Institute of Translational Pharmacology, Consiglio Nazionale delle Ricerche (C.N.R.), 00133 Rome, Italy

**Keywords:** human immunodeficiency virus, reverse transcriptase, in vitro transcription, quantitative PCR assay

## Abstract

Based on previous experience in our laboratory, we developed a real-time reverse transcriptase (RT) quantitative PCR (RT-qPCR) assay for the assessment of very low levels of HIV-1 RT activity. The RNA, acting as a template for reverse transcription into cDNA by HIV-1 RT, consisted of a synthetic RNA ad hoc generated by in vitro transcription and included a coding sequence for HSV-1 gD (gD-RNA-synt). Different conditions of variables involved in the RT-qPCR reaction, notably different amounts of gD-RNA-synt, different mixes of the reaction buffer, and different dNTP concentrations, were tested to optimize the assay. The results indicated that the gD-RNA-synt-based RT assay, in its optimized formulation, could detect a specific cDNA reverse transcription even in the presence of 1 × 10^−9^ U of HIV RT. This achievement greatly improved the sensitivity of the assay over previous versions. In summary, this constructed RT-qPCR assay may be considered a promising tool for providing accurate information on very low HIV-1 RT activity.

## 1. Introduction

Assays based on the evaluation of the activity of the RNA-dependent DNA polymerase, the reverse transcriptase (RT) enzyme that characterizes all retroviruses [1,2], were proposed early on to appraise the replication capacity of HIV-1 viral particles [3]. In addition to earlier assays, based on the incorporation of radiolabeled nucleotides, a number of nonisotopic methods have been successively described for the assessment of RT activity [4]. Notably, a heterologous RNA as a template, the polymerase chain reaction (PCR), and various techniques for detection characterized most of this second generation of assays for the assessment of RT activity [5,6,7,8,9,10]. Interestingly, aside from being employed as a surrogate to estimate infectious HIV-1 [11], these methods were also adopted to assess the inhibitory activity of compounds towards HIV RT [12,13,14]. Moreover, due to the reliability of their results, RT-based assays have been proposed as a good alternative choice for monitoring patients living with HIV (PLWH) in resource-limited countries [15,16]. Regarding the sensitivity of HIV RT detection—increasingly considered as a relevant requirement for these assays—the multiplicity of the technical variants has enabled its progressive improvement over the years [17,18]. In fact, the introduction of anti-HIV antiretroviral therapy (ART) and its refinement over the years has dramatically changed the course of the disease in HIV-1 patients, changing AIDS to a chronic health issue [19]. Nevertheless, it was soon realized that HIV-1 stably persisted in the cellular reservoirs of ART-treated patients, and treatment interruption invariably led to viral rebound [20,21,22]. The characteristics and physiopathological and therapeutic significance of the cellular reservoir and persisting virus in naive or ART-treated patients living with HIV (PLWH) are complex issues, including various and interacting aspects, which have been deeply investigated over the years [23,24,25,26,27,28,29,30,31,32,33]. Most of the pivotal aspects concerning HIV-1 persistence have been only partially clarified. Nevertheless, it seems clear that a minority of the reservoir clones are proviral transcriptionally active and, where carrying intact provirus, produce infectious virions. Moreover, a transition from latency to the active phase could lead to the viral blips occasionally observed in ART patients [34,35]. The importance of defining the extent of the HIV-1 persistence has induced the development of even more informative methods to assess the HIV reservoir [36,37,38,39,40,41,42,43].

We have recently demonstrated the potentialities of a real-time RT quantitative PCR assay to quantitatively measure the enzymatic activity of HIV-1 RT directly at the plasmatic level to screen for the functional status of HIV-1 in PLWH [44]. This assay was derived from previously described methods for screening anti-HIV-1 candidate drugs or evaluating RT activity in samples from HTLV-1-infected ATL patients [45,46]. However, a limit reported in the study was that the formulation of the assay would not properly estimate RT activity in patients with low VL levels [44]. This induced us to develop a method with improved sensitivity. For this purpose, each single variable factor inherent to the protocol of the assay previously published by us has been subjected to a targeted revision. The results we obtained show that the major innovations introduced allowed the detection of a specific cDNA reverse transcription even in the presence of extremely diluted amounts of HIV-1 RT. Thus, such a constructed RT-qPCR assay may be considered as a potential new tool for providing accurate information on very low levels of HIV-1 RT activity for preclinical and clinical requirements.

## 2. Materials and Methods

### 2.1. Cells

Stable transfected I143tk- cells, ectopically expressing the US6 gene of herpes simplex virus 1 (HSV-1) and encoding for glycoprotein D (gD), already available at our laboratory, were cultivated as previously described [47]. These cells, named I143-J3, directly utilized in previous assays as a source of total RNA template [12,45,46], were differently used in this new assay as a source of DNA template for the amplicon synthesis by PCR reaction.

### 2.2. Generation of the RNA Template by In Vitro Transcription

The main strategy pursued during the present study was the substitution of total RNA extracted from cells stably expressing the HSV-1 gene for gD with a synthetic RNA containing genetic information for the same gene (gD-RNA-synt) as a template for the reverse transcription reaction. For this purpose, three main operational phases were carried out: (i) generation of amplicons containing a portion of the US6 gene for gD and the promoter sequence for the T7 RNA polymerase to utilize as DNA templates for in vitro transcription, (ii) in vitro transcription by T7 RNA polymerase, and (iii) purification of the generated specific gD-RNA-synt molecule. To prepare the DNA template containing the T7 promoter sequence upstream to the initial portion of the coding sequence for the US6 gene (GeneBank accession number L09242.1.), I143-J3 cells, controlled for HSV-1 gD expression by indirect immunofluorescence using a specific anti-gD antibody (DL6, Santa-Cruz), were seeded at a density of 5 × 10^5^ cells/well. After 24 h of incubation at 37 °C, the cells were trypsinized, washed with PBS, and collected in ice for total DNA extraction using the commercial Quick-DNA Microprep Plus Kit (Zymo Research). The obtained total DNA, containing the expressing vector for gD, identified as pcDNA-J3, was quantified by a NanoDrop 2000 Spectrophotometer (Thermo Scientific, Waltham, MA, USA) and then amplified with the T7 promoter primer (T7pw) and the gDc9Rev primer. As a control, samples without the template (no template control, NTC) were used. In addition, a parallel amplification was set up using the oligo gDc9Fw designed on a US6 internal sequence as a forward primer. The sequences of the selected primers and the expected sizes for the related amplicons are shown in Table 1.

The primers were designed to obtain a maximum amplicon size of 650 bp using Primer-BLAST (http://www.ncbi.nlm.nih.gov/tools/primer-blast/, accessed on 15 March 2022). The options returned by Primer-BLAST were analyzed for their structural and thermodynamic characteristics using NetPrimer software (http://www.premierbiosoft.com/netprimer/, accessed on 15 March 2022) and OligoFaktory (http://oligofaktory.lanevol.org/index.jsp, accessed on 8 September 2017) and then controlled for their specificity by means of single BLAST (Blast-Like-Alignment-Tool, (http://www.ncbi.nlm.nih.gov/BLAST/, accessed on 15 March 2022). PCR amplification was performed in 25 μL with 200 nM of each primer, 0.25 mM dNTP mix, and 1×PCR buffer with 1.5 mM MgCL2 and 0.5 U of AmpliTaq Gold™ DNA polymerase (Thermo Fisher Scientific, Waltham, MA, USA Cat no. 4398813). The PCR conditions were 95° for 10 min, followed by 35 cycles of 95° for 15 s, 58° for 30 s, and 72° for 45 s. The PCR products were run on a 1.5% agarose gel to confirm the size of the amplicons. Next, in vitro transcription was performed with the MEGAScript T7 Transcription kit (Thermo Fisher Scientific, Cat no. AM1333). Each 20 μL reaction contained 2 μL of 10×buffer, ATP, CTP, GTP, UTP, T7 RNA polymerase, and 100 ng (~2 µL) of T7-tagged amplicon. After incubating at 37° for 3 h, the DNA was digested with 2 U of TURBO-DNase at 37 °C for 15 min. The in vitro transcribed RNA was subsequently purified by a MEGAclear kit (Ambion Cat no AM1908), eluted in 30 μL of DEPC-dH2O, aliquoted, and stored at −80 °C. As a control, in parallel, a reaction was set up with 1 µg of linearized pTRI-Xef plasmid, supplied in the kit. Quantitation of the RNAs was performed spectrophotometrically at 260 nm by the NanoDrop 2000. The gD-RNA-synt concentration was measured in duplicate, and the concentration was then converted to the molecule number using Avogadro’s number.

Finally, to verify the expected sizes, the in vitro transcribed RNAs were visualized on a denaturing 5% acrylamide/8M urea gel in TBE 1x buffer, adding 10 µg/mL ethidium bromide to the RNA sample before loading the gel.

### 2.3. Overview and Methodology of the RT-qPCR Assay

The developed assay is a two-step RT-qPCR assay. The first step of the assay is a primer-targeted generation of a cDNA strand specific for a portion of the HSV-1 gD gene by part of HIV-1 RT. This step used gD-RNA-synt, generated in house by in vitro transcription, as a template. The second step consists of the quantitative evaluation of the reverse transcribed cDNA, through a real-time quantitative PCR reaction. The technical procedures carried out during the first and the second steps of the assay were similar to those previously described by us, except for the source of the RNA template, the concentrations/characteristics of some reaction reagents, and other variables involved in the procedural protocol, such as the times utilized in the different phases. The final protocol of the assay was chosen based on the best procedural conditions, taking into account the primary objective to ameliorate the sensitivity of RT detection. In particular, the selected conditions were the ones that resulted in the lowest Ct values in the optimization experiments. Considering that (i) the basic procedures have been described in detail in previous publications and (ii) the various variables utilized during the selection phases are described in detail in the “Results”, the methodology of the RT-qPCR assay, in its optimized protocol, will be only briefly described here.

#### 2.3.1. First Step, Reverse Transcription of gD-RNA-synt

Synthesis of cDNA was performed from gD-RNA-synt as a template using different amounts of a commercial recombinant HIV-1 RT (Calbiochem, Merck KGa, Darmstadt, Germany). Specifically, 10^6^ molecules of in vitro transcribed RNA were mixed with 1 µL of 10× homemade RT Buffer, named “TriB 1.5” (10 mM Tris HCl, 50 mM KCl, 1.5 mM MgCl2, 0.1% Triton), 500 nM of gD-specific reverse primer (5′-GCATTCGGTGTACTCCATGAC-3′), 0.1 mM of each dNTP, and 1 µL of the stated dilution of HIV RT in a total volume of 10 µL. The mixture was set up on ice and then incubated for 240 min at 37 °C and 5 min at 85 °C. The resulting cDNA was stored at −20 °C.

#### 2.3.2. Second Step, SYBR Green qPCR

In the second step of the assay, real-time quantitative PCR was performed on an Applied 7500 System instrument. Amplification of specific PCR products was detected using the Maxima SYBR Green/ROX qPCR 2X Master Mix (Thermo Scientific, Cat. no K0222). Each real-time PCR reaction was performed in MicroAmp 96-well PCR plates (Applied Biosystems™) in a total reaction volume of 25 μL containing 1 X SYBR Green Mix, 10x diluted Rox solution, 0.5 U of UDG (Thermo Scientific) 300 nM forward (gDFw, 5′-CCGGAAACAACCCTACAACC-3′) and reverse (gDRev, 5′-GCATTCGGTGTACTCCATGAC-3′) primers, and 1 µL of the first step dD-specific cDNA product. PCR cycling was performed as follows: 2 min at 50 °C (UDG pretreatment), 10 min at 95 °C, followed by 40 rounds of 15 s at 95 °C and 60 s at 60 °C. A melting curve (65–95 °C; at increments of 0.5 °C) was generated to verify the specificity of the primer amplification.

### 2.4. Statistical Analysis

To monitor the possible sampling error and experimental error, three experiments with three technical qPCR replicates for each RT sample were independently performed, unless otherwise indicated. Quantifications shown in the figures represent the mean values and standard deviation (S.D.) of the means calculated for the representative experiments shown in the graphics. Nonlinear regression analysis was performed using GraphPad Prism v.6.01 software (GraphPad Software, Inc., La Jolla, CA, USA).

## 3. Results

### 3.1. Synthesis of a US6-Based RNA Template by In Vitro Transcription and Its Validation

**Figure 1:**
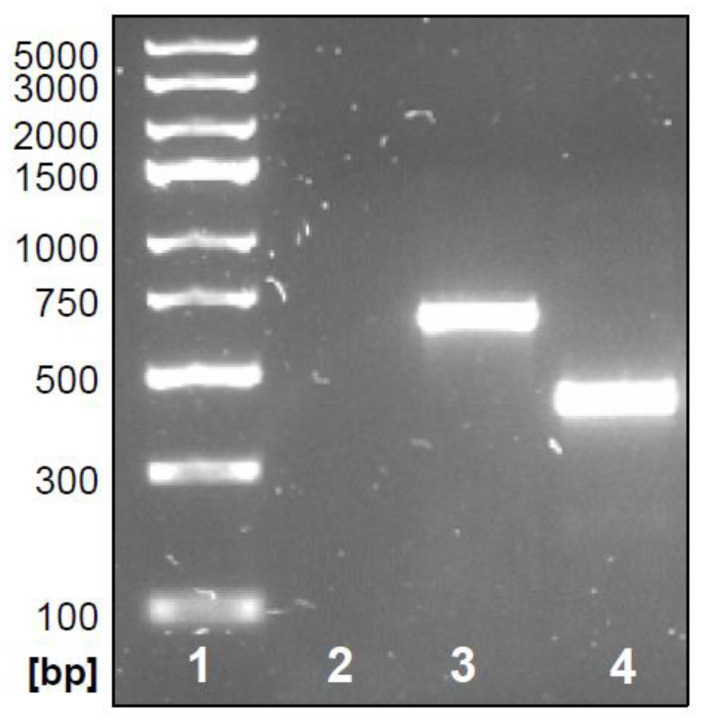
Agarose gel analysis of the obtained PCR products. Lane 1: DNA ladder; lane 2: no template control (NTC); lane 3: amplicon from T7pFw and gDc9Rev primer pair; lane 4: amplicon from gDc9Fw and gDc9Rev primer pair.

The main objective of the present study was the development of an ultrasensitive assay that could be utilized for the analysis of low levels of HIV-1 RT enzymatic activity. The actual technical challenge was detecting the retrotranscriptional activity of HIV-1 RT by an RT-qPCR assay, even when the enzyme was present in minimal quantities in the reaction mixture. Moreover, a mandatory requirement was the simplicity of execution that characterized the two-step RT-qPCR assay previously drawn up by us. In the previously described assay, total RNA extracted from human cells stably transfected with the US6 gene for gD of HSV-1 was utilized as the source of the exogenous RNA template to be retrotranscribed. Differently for the new assay, a specific RNA molecule, drawn on the sequence of a portion of the same US6 HSV-1 gene, was generated in vitro as the RNA template for the reverse transcription reaction. In fact, the presence of a unique RNA species in the reaction mixture was considered a favorable condition in the case of very low RT concentrations. For this purpose, a DNA template containing a portion of the selected gene and provided with the T7 promoter sequence for the RNA polymerase was generated. The design and choice of the oligonucleotide sequences to be used took into account the following conditions: (i) the portion of the US6 gene to be amplified should not be less than 400 bp or greater than 800 bp to remain in an optimal length range for in vitro transcription using T7 RNA polymerase; (ii) the region to be amplified had to contain the portion of US6 recognized by the primer pair already used for the previously validated SYBR Green RT-qPCR to allow a direct comparison between the previous conditions and the current strategy; and (iii) the presence of the T7 promoter upstream of the specific US6 sequence located should be recognized by the T7 RNA polymerase. The pcDNA 3.1 vector carrying the US6 gene for gD of the I143-J3 cells [48] included the T7 promoter region upstream of the insertion point of the sequence coding for gD by itself (Figure S1). Then, a forward primer (T7pFw) upstream of the molecular cloning site of the T7 promoter and a reverse primer (gDc9Rev) inside the sequence coding for gD of pcDNA 3.1 was designed. As a control for the specific T7 promoter-carrying amplicons, an additional forward primer (gDc9Fw) targeting an internal US6 sequence was designed (see Table 1 and Figure S1). Total DNA from I143-J3 cells was then extracted and used to set up the PCR reactions in the presence of different combinations of the described primers and according to adequate reaction conditions. At the end of the reaction, 5 µL for each sample was loaded into a 1.5% agarose gel and stained for analysis. The results showed the specific expected sizes for the corresponding amplification products (Figure 1, lanes 3 and 4). Lane 2 was a negative control of the PCR reaction (no template control, NTC). Based on these results, amplicon 3 obtained with the pair of primers T7pFw + gDc9Rev was used as a DNA template for the next stage of in vitro transcription.

**Figure 2:**
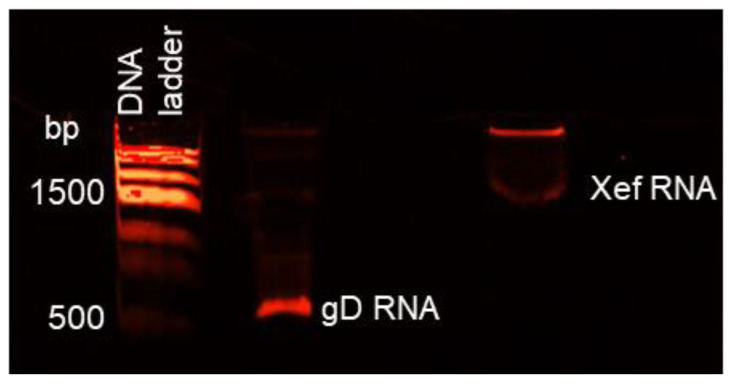
Acrylamide/Urea gel analysis of RNA produced by in vitro transcription.

A volume of 2 μL of the PCR reaction was added as the DNA template in a volume of 20 μL in vitro transcription reaction set up with the bacteriophage T7 RNA polymerase and reagents from a commercial kit. In parallel, a linearized pTRI-Xef plasmid supplied in the kit was transcribed as the reaction control. Following the in vitro transcription, DNase was added to digest the DNA template, and the mixtures were purified by affinity RNA columns. Then, the in vitro transcribed RNA molecules corresponding to the selected region of the US6 gene for HSV-1 gD (gD-RNA-synt) were further analyzed by a 5% acrylamide/8M urea denaturing gel. As shown in Figure 2, both the gD-RNA-synt and the Xef-RNA control showed the expected sizes, at about 530 b and about 1.89 Kb, respectively. To ensure that possible contamination from the DNA template could not affect the subsequent phases of the assay, further DNA digestion and purification steps were performed before using the gD-RNA-synt.

To validate the possible use of the gD-RNA-synt molecule as a template, this synthetic RNA was utilized in real-time RT-qPCR reactions carried out according to the already published protocol for a real-time RT-qPCR assay to quantify HIV RT activity [46]. Moreover, we wanted to verify whether the purified specific gD-RNA-synt as a template could improve by itself the performance of the assay with respect to that obtained using total RNA from cells overexpressing the gD gene as a template. For this purpose, a comparison was made between the amplification curves of the two assays by exclusively varying the initial source of RNA template and its amount (10 ng of gD-RNA-synt versus 150 ng of total RNA, as previously used) and using a fixed amount of 0.025 U of HIV RT. The results showed a threshold cycle (Ct) mean value of about 3–4 when gD-RNA-synt was used as a template in comparison with a mean Ct value of about 21–25 when total RNA was utilized in the reaction mixture. The results clearly indicated the improved efficiency of the synthetic gD-RNA-synt-based assay (Table S1). The following step for the validation of the gD-RNA-synt-based assay was the optimization of the amount of gD-RNA-synt to be used. Starting from a solution containing 10^9^ molecules/μL and corresponding to 300 pg/μL of purified gD-RNA-synt, three tenfold dilutions were prepared and tested as a volume of 1 μL each. Samples containing the different amounts of gD-RNA-synt were incubated at 37 °C for 1 h in the presence of 1 × 10^−2^ U of HIV RT, according to the retrotranscription protocol. As a control, samples without the RT enzyme were set up. At the end of the retrotranscription reaction, 1 μL of the produced cDNA was used for real-time PCR quantification. The results indicated that using gD-RNA-synt as a template enabled obtaining good quantities of retrotranscribed cDNA even in the presence of 1 × 10^−2^ U of HIV RT and that using an amount of 10^7^ or 10^6^ molecules/μL of gD-RNA-synt in the mixture resulted in no amplification in the corresponding RT-minus samples. Using, instead, an amount of 10^8^ molecules/μL of gD-RNA-synt occasionally gave an amplified DNA at very high threshold cycles, in repeated tests in the RT negative controls (Figure S2). This was presumably attributed to contaminating DNA still present in the gD-RNA-synt in extremely low quantities but detectable only by greatly increasing the amount of gD-RNA-synt. Consequently, an amount of 10^6^ molecules/μL of gD-RNA-synt was utilized in the successive steps of optimization.

**Figure 3:**
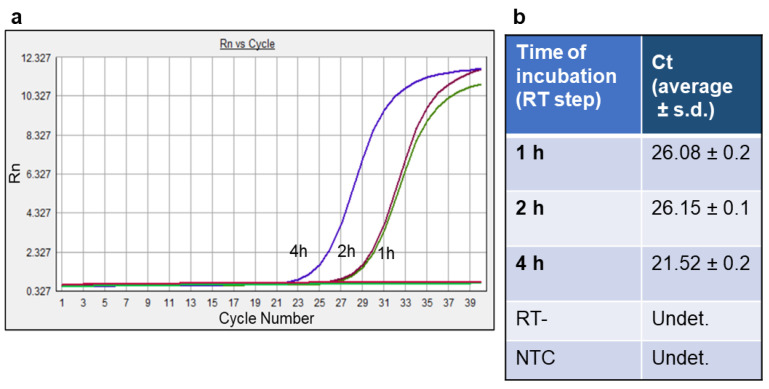
Effects of three different incubation times during the retrotranscription phase on the amplification curves (**a**) and Ct values (**b**) of the cDNA real-time PCR analysis. RT-, negative control in the absence of the HIV RT enzyme. NTC, and negative control in the absence of the template in the RTqPCR reaction.

To define whether the time of the retrotranscription phase of the assay, performed using gD-RNA-synt as a template, could affect the Ct values obtained, experiments were conducted in which three different retrotranscription times were examined. Figure 3 shows the amplification curves obtained in a representative experiment by incubating 10^6^ gD-RNA-synt molecules in the presence of 10^−2^ U of HIV RT for 1 h, 2 h, and 4 h at 37 °C. Following a further incubation for 5 min at 95 °C, the generated cDNAs were quantified by SYBR Green PCR, according to the general protocol. The results showed that an incubation time of 2 h did not create substantial improvements in the activity of retrotranscription compared to the incubation of 1 h. In contrast, the prolongation to 4 h of the retrotranscription reaction allowed the generation of greater quantities of cDNA, indicating that a single hour of incubation was not sufficient to highlight a fully completed reverse transcription activity using gD-RNA-synt at the selected reaction concentration. Thus, this aspect was also taken into consideration in the successive phases of the setting up of the assay.

**Figure 4:**
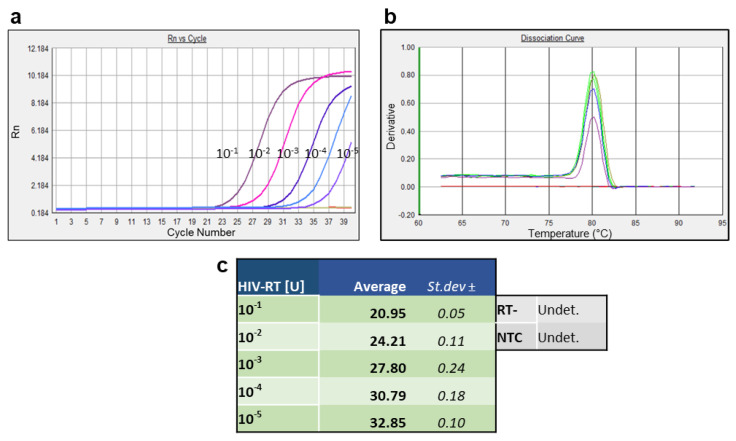
Amplification (**a**) and dissociation (**b**) curves and Ct values (**c**) of real-time PCR analysis of cDNAs produced using tenfold dilutions of the HIV RT enzyme, up to 1 × 10^−5^.

The final step for the validation of the gD-RNA-synt as a template for an RT-qPCR assay to quantify HIV RT was the evaluation of its efficiency in correctly detecting scalar dilutions of the HIV RT enzyme, using the protocol optimized thus far for the retrotranscription phase. Five tenfold dilutions of the HIV RT enzyme up to 10^−5^ U were used for retrotranscription reactions performed with 10^6^ gD-RNA-synt as a template according to a thermal protocol of 4 h at 37 °C and 5 min at 95 °C. The cDNA obtained was subjected to real-time PCR amplification using SYBR Green dye. In these reaction conditions, the new gD-RNA-synt achieved the dilution of 10^−5^ U of the HIV RT enzyme while maintaining an RT-specific threshold cycle value in all replicates (Figure 4a). Note that the tests performed according to the protocol using total RNA as a template resulted in a maximum of 2.5 × 10^−3^ U of HIV RT enzyme with RT-specific activity in all replicates (Table S2). These results clearly demonstrated that the modifications made in the retrotranscription phase were able to increase the sensitivity of the enzyme-specific assay. Moreover, the absence of fluorescence in the RT control samples indicated that no trace of contaminating DNA was present in the reactions. In addition, analysis of the threshold cycles indicated that relatively low and HIV RT dose-dependent average Ct values could be found, confirming a good sensitivity (Figure 4c). Finally, the melting temperature analysis indicated that the products were highly specific (Figure 4b).

### 3.2. Optimization of the RT Reaction Conditions to Improve the Sensitivity of Detection of the HIV-1 RT Assay

To improve the sensitivity of the assay, the technical phases leading to the detection of the amplified cDNA were then considered. The amplification efficiency of the real-time PCR reaction was assessed using the linear relationship between the Ct value and the decimal logarithm of the number of copies of the template, according to the proper procedure [49]. For this purpose, experiments were performed using amplification mixtures with serial dilutions of the retrotranscribed cDNA to detect the corresponding Ct values (Table 2).

**Figure 5:**
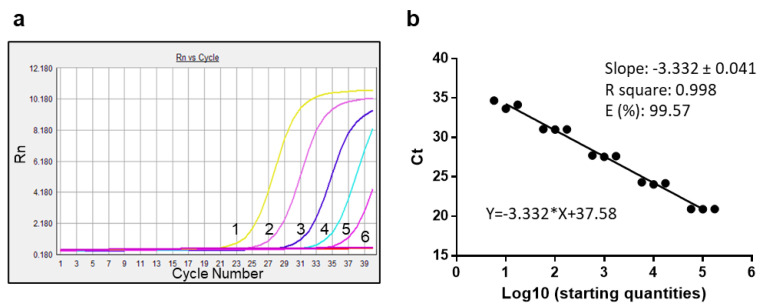
SYBR Green real-time PCR amplification efficiency. (**a**) Amplification curves obtained from tenfold dilutions of the cDNA corresponding to 10^5^ molecules of template RNA. Identification numbers reported in Table 2. (**b**) Serial dilutions of the cDNA template, corresponding to the identification numbers reported in Table 2, were used and assayed in triplicate. Each black circle refers to one of these assessments. The resulting Ct values are plotted against the Log10 of the starting quantity. Calculation of the slope, R2, and efficiency, are also reported.

As summarized in Figure 5, in a representative experiment, the amplifications of five tenfold dilutions of cDNA resulting from reverse transcription of 10^6^ molecules of gD-RNA-synt obtained a curve with a slope equal to 3.33. This value corresponded to an efficiency value close to 100%. The R-square coefficient >0.99 was also indicative of the excellent performance of the set of primers used for PCR and of the robustness of the data obtained. Overall, these data indicated that the amplification phase in SYBR Green real-time PCR did not require further optimization.

**Figure 6:**
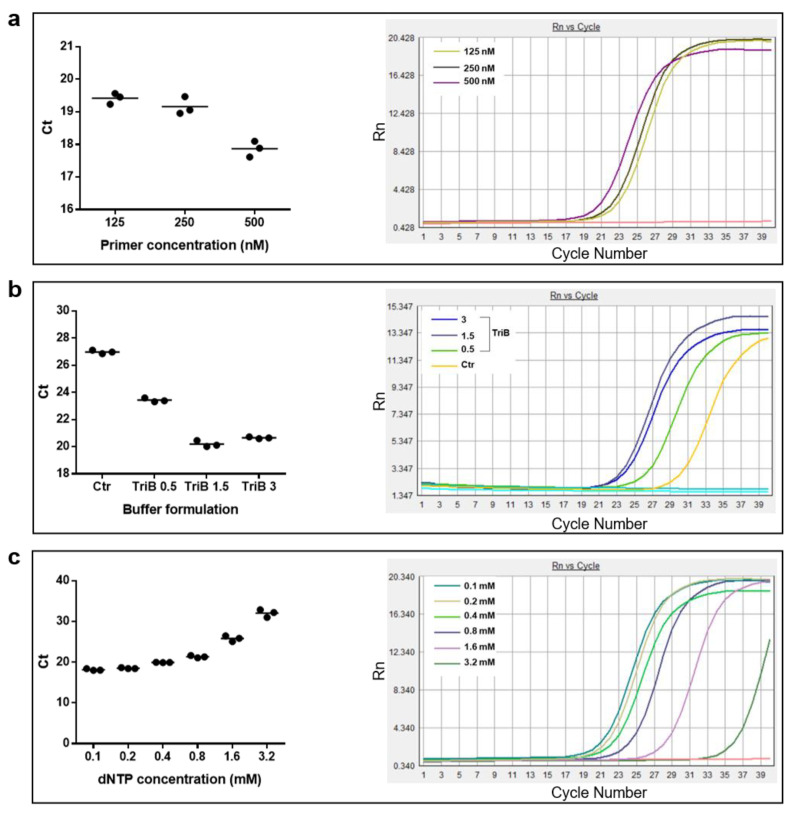
Effects of different components of the retrotranscription reaction on the outcome of the RT-qPCR assay performed under the following fixed conditions: gD-RNA-synt = 10^6^ molecules, HIV-1 RT = 1 × 10^−3^ U, incubation time = 4 h. (**a**) Effects of three different gD-specific primer concentrations (125, 250, and 500 nM) on triplicate Ct values (left panel; line = mean values) and on the amplification curves (right panel). (**b**) Effects of three different formulations of the reaction buffer (TriB 0.5; TriB 1.5; TriB 3) on the triplicate Ct values (left panel; line = mean values) and on the amplification curves of one representative experiment (right panel). Ctr = 10 mM Tris HCl, 50 mM KCl, 1.5 mM MgCl2; TriB 0.5 = 10 mM Tris HCl, 50 mM KCl, 0.5 mM MgCl2, 0.1% Triton; TriB 1.5 = 10 mM Tris HCl, 50 mM KCl, 1.5 mM MgCl2, 0.1% Triton; TriB 3 = 10 mM Tris HCl, 50 mM KCl, 3.0 mM MgCl2, 0.1% Triton. (**c**) Effects of six different concentrations of dNTPs from 0.1 mM to 3.2 mM on triplicate Ct values (**left** panel; line = mean values) and on the amplification curves of one representative experiment (**right** panel). Red lines in (**a**,**c**), and blue line in (**b**) refer to negative controls.

In the attempts to detect the smallest possible amount of RT activity, a series of experiments were then performed in which different conditions of a single component of the first phase of the assay, i.e., the retrotranscription reaction, other than the gD-RNA-synth template and the incubation time, were assayed. In this round of experiments, different concentrations of the specific RT primer (Figure 6a), different formulations of the reaction buffer (Figure 6b), and different amounts of the deoxyribonucleotide triphosphate (dNTPs) were tested. The results led to the identification of the optimal reaction conditions for the abovementioned three factors, as 500 nM for the concentration for the primer, as Triton 0.1 mM and MgCl2 1.5 mM % for the buffer, and to an amount of 0.1 or 0.2 mM for the dNTPs, as shown by the amplification curves located to the left side of the graphs (Figure 6).

**Figure 7:**
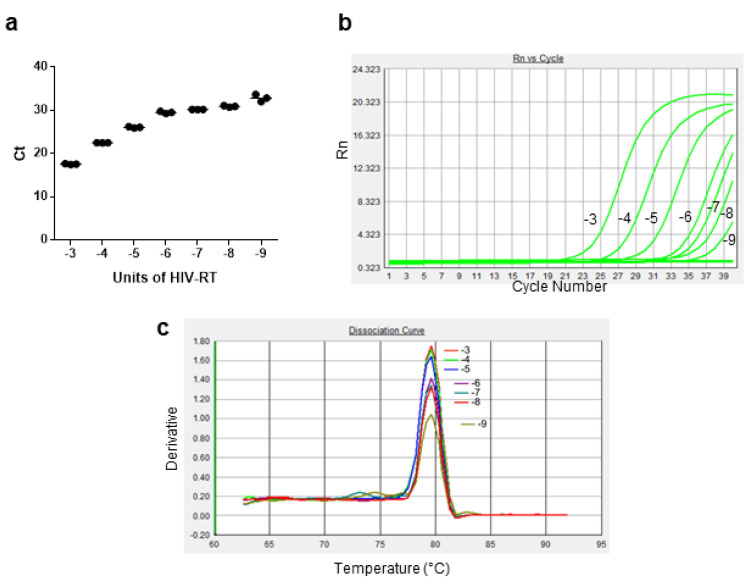
Effects of different amounts of HIV-1 RT on the outcome of the optimized RT-qPCR assay. Main reaction conditions: gD-RNA-synt = 10^6^ molecules, RT-primer = 500 nM, dNTPs = 0.1 mM, incubation time = 4 h. (**a**) Plot graph of triplicate Ct values (line = mean values) versus the HIV RT quantities. (**b**) Amplification curves and (**c**) dissociation curves obtained with HIV-1 RT amounts ranging from 10^−3^ to 10^−9^ U. Graphics and data refer to one representative experiment.

Thus, putting together the information collected during the different experimental phases of the study, the final step was to verify the threshold quantity of HIV-1 RT detectable with the optimized assay. For this purpose, a series of experiments were performed using 10^6^ molecules of gD-RNA-synth as a template, 500 nM RT-primers, the selected reaction buffer, 0.1 mM dNTPs as reaction mixture, 4 h incubation for the amplification phase, and serial dilutions from 1 × 10^−3^ to 1 × 10^−9^ U of HIV-1 RT. As shown in Figure 7a, the identified conditions obtained amplification curves up to the samples in which an amount of 1 × 10^−9^ U of HIV-1 RT was added to the reaction mixture. In addition, the melting temperature analysis and the corresponding dissociation curves indicated that the amplified products were highly specific (Figure 7c).

## 4. Discussions

The present study reports the technical details of the setting up of an assay to provide information on the enzymatic activity of HIV-1 RT, even in the presence of small amounts of the viral enzyme. Our study was carried out taking into account our previous experience in the specific fields [12,45,46]. To reach the goal of high sensitivity, the strategy adopted mainly consisted of making fundamental adjustments to the previous assay utilized by us to detect plasmatic RT activity in PLWH, which was not endowed with the required sensitivity [44]. In accordance with the previous assay, we thought that an RNA sequence typical of a viral genome might represent an optimal source to be supplied as a template to HIV-1 RT, since, during their evolution, different viruses often maintain structural characteristics (for example, the percentage of composition in nitrogenous bases and the consequent tendency to form secondary structures) from which the cellular genomes slightly deviate. These are characteristics that could be useful to “replicate” with respect to the activity carried out by an enzyme that is also viral, especially to ensure its optimal performance. In addition, considering that the ultimate aim of our study was the setting up of an assay appropriate to detect HIV-1 RT activity in plasma samples from PLWH, we had to necessarily exclude the RNA template from HIV-1 genes or from other RNA viruses potentially detectable in the blood of the same individuals, as well as from human genes. This could be an unavoidable limitation of our assay. Then, we generated an RNA template by in vitro transcription from an exogenous template of a DNA virus. Thus, our choice was the substitution of total RNA extracted from stably transfected cells harboring the US6 gene for gD of HSV-1, utilized in our previous version of the assay, with a synthetic RNA molecule covering part of the same gene. Indeed, this choice could also meet the requirements of (i) an already well-experienced source of RNA template for cDNA synthesis by HIV-1 RT, and (ii) an RNA template useful to verify an improved sensitivity to the new version of the assay with respect to the previous one by direct comparison. Further amelioration of the limit of RT detection was achieved by adjusting the concentrations of the reagents and the composition of the reaction buffer. Altogether, the modifications made allowed us to reach a reasonable limit of detection in terms of the units of commercial HIV-1 RT detectable in the optimized assay. The sensitivity of assays to detect RT activity has been greatly improved over the years. In fact, the authors of articles focused on this subject concluded that the more advanced assays should be capable to detect RT amounts equivalent to a few HIV-1 virions. In fact, the exact calculation of virion equivalence for RT units or RNA molecules is not a simple matter and could be highly influenced by the methodologies utilized for enhancing RT detection, the reference standard, and the source of RT utilized. In particular, it seems very difficult to exactly predict the limit of detection of an RT assay when plasmatic HIV-1 RT from PLWH is utilized as a source of the enzyme instead of pure recombinant RT. This is why, in this study, devoted to the setting up of a new assay by modifying most of the variables involved in the reaction mixture, a unique well-standardized source of HIV-1 RT was utilized. Moreover, even when particular attention was paid by different research groups during the setting up of the various assays to simplify them as much as possible, the technical demands, labor intensity, and expense were not always limited. In contrast, the new assay described here has all the features of a very simple real-time RT quantitative PCR laboratory test. The sensitivity we obtained in the best conditions for our assay seems slightly lower than that reported by other authors. In fact, the RT detection limit of other PCR-based assays, calculated in terms of corresponding HIV virions, was approximately one viral particle, with a Ct value of about 38 [18]. In contrast, in our case, the limit of RT detection, calculated using the same formula utilized by other authors (3.36 × 10^−10^ = 1 HIV-1 particle), corresponds to the RT present inside about three virions. Nevertheless, there are many uncontrollable factors that could influence an RT-based real-time PCR reaction working at its limit of detection. In fact, even in our experiments not all the replicated samples, during the various phases of the setting up of the assay, ensured exactly the same limit of detection. This is the reason why in this phase of the study, different to other authors, we preferred to limit our experiments to a maximal dilution of HIV-1 RT to 10^−9^ U to avoid exceeding the limit of an average Ct of 35 to evaluate the reliability of the assay. All this makes it possible to assert that the new assay has high sensitivity and a very simple and quick execution mode.

Information on the replication-competent status of residual viremia is a main objective in the management of PLWH treated with ART. In fact, the main purpose of antiretroviral therapy is a lasting suppression of viral replication up to potentially undetectable plasmatic levels. This end point should ensure the delay of the possible recrudescence of the disease and to prolong the life expectancy and quality of life of PLWH. The availability of increasingly sensitive assays capable of effectively detecting even small amounts of replicating viruses is, therefore, a fundamental aspect of current anti-HIV strategies. Among the different assays, the amplification of the RNA of the HIV virions present in the serum represents the most widespread and validated strategy. This method, however, while representing the gold standard assay for the evaluation of VL, might have some limitations in providing, by itself, exact information on the real replicating status of residual or blipping viremia. In fact, comprehensive information on the replication-competent status of the HIV-1 reservoir in PLWH is far from being easily ascertained. Thus, detection of HIV-1 RT activity even in the presence of small amounts of the viral enzyme could possibly have the requirements to meet these needs.

In conclusion, the RT-qPCR assay described here, using a gD-RNA-synt as a template for the first time, may be considered right now as an additional tool for basic or translational studies devoted to the accurate evaluation of very low levels of HIV-1 RT. Among these, of particular interest are those aimed at selecting new antiviral strategies by screening even more effective and tolerable compounds in vitro. Moreover, the assay could also represent a promising basis for furnishing information on the functional virological status of PLWH. In fact, the results showed that the RT detection limit of the new version of our assay corresponds to the amount of enzyme contained in three viral particles. Future studies, however, using plasmatic samples from HIV-1 infected patients as a source of RT are necessary. Only the results of these studies will confirm whether this RT-qPCR assay may represent a valid methodological optimization for the detection of small quantities of HIV RT enzyme in PLWH.

## Figures and Tables

**Table 1:** Sequences of the primers selected for the amplicon synthesis.

Primer Identification	5′ > 3′ Sequence	Amplicon Size (bp)
T7pw	GAAATTAATACGACTCACTATAGGGAGA	568
gDc9Rev	CCCAGGTTATCCTCGCTGAC	
gDc9Fw	CTTTCGCGGCAAAGACCTTC	403
gDc9Rev	CCCAGGTTATCCTCGCTGAC	

**Table 2:** Serial dilution of the cDNA template and corresponding identification number.

Identification Number	Dilution	Starting Quantity per Reaction (Copies cDNA)
1	Undiluted	10^5 1^
2	1:10	10^4^
3	1:100	10^3^
4	1:1000	10^2^
5	1:10,000	10^1^
6	1:100,000	10^0^

^1^ One µL of each sample (1–6) was used in the qPCR. Assuming a complete transcription into cDNA, this corresponds to a final concentration of 10^5^ copies/µL cDNA for the undiluted sample.

## Data Availability

The data that support the findings of this study are available in the text and in the supplementary material of this article or from the corresponding author upon reasonable request.

## Supplementary Materials

The following supporting information can be downloaded at: https://www.mdpi.com/article/10.3390/life12081130/s1.
