# Supplementary material for: Quantitative Evaluation of Very Low Levels of HIV-1 Reverse Transcriptase by a Novel Highly Sensitive RT-qPCR Assay
Source: Life (Basel). 2022 Jul 27;12(8):1130. doi: 10.3390/life12081130 (PMC9410348; doi:10.3390/life12081130)
Supplement: Supplementary file 1 [file life-12-01130-s001.zip › life-1768441-supplementary.pdf]

Supplementary Materials

for

Quantitative evaluation of very low levels of HIV-1 reverse transcriptase by a highly sensitive RT-qPCR assay

by Francesca Marino-Merlo, Valeria Stefanizzi, Agnese Ragno, Lucia Piredda, Sandro Grelli, Beatrice Macchi, Antonio Mastino

Contents

Supplemental Figure S1

Supplemental Figure S2

Supplemental Table S1

Supplemental Table S2

Supplemental Figure S1

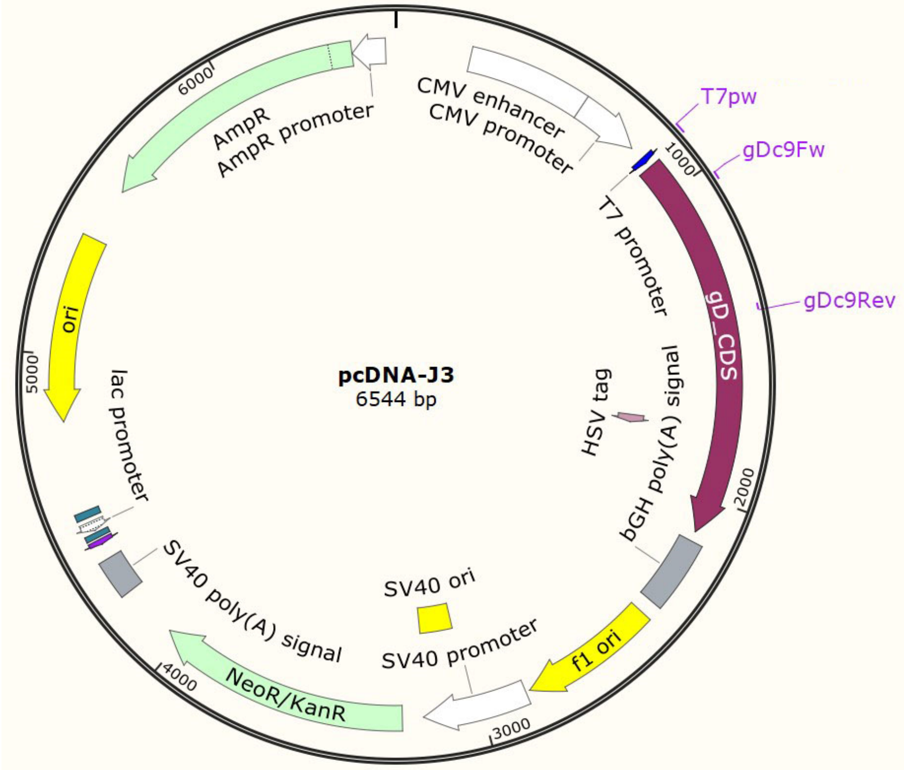

Figure S1. The pcDNA-J3 expression vector. Schematic drawing of pcDNA-J3 expression vector contained in I143-J3 cells. The coding DNA sequence (CDS) of HSV-1 US6 gene in pcDNA 3.1 expression vector, corresponding to GenBank accession number L09242.1, is depicted as the dark red region gD-CDS inserted downstream of T7 promoter region (small blue arrow). Position and orientation of the primers used is represented by the violet lines (see Table 1 for amplicon sizes). The map is drawn with SnapGene® software (from Insightful Science; available at snapgene.com).

Supplemental Figure S2

(a)

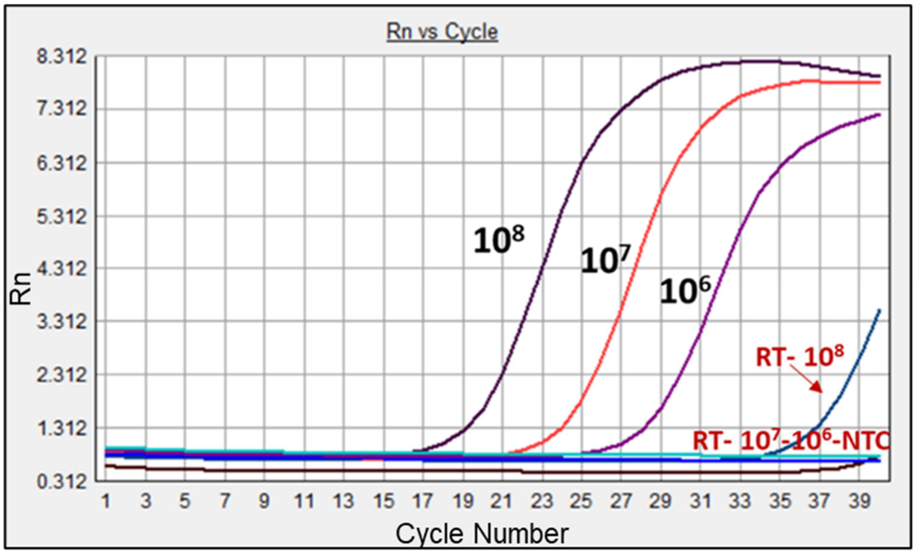

(b)

gD-RNA-synt (number of molecules used for RT reaction)	RT+		RT-			
	CT average	*St.dev ±*	Ct_{v1}	Ct_{v2}	Ct_{v3}	Ct_{v4}
**10^8**	17.22	0.04	38.4	Undet	34.4	Undet
**10^7**	21.65	0.05	Undet	Undet	Undet	Undet
**10^6**	26.02	0.04	Undet	Undet	Undet	Undet

Figure S2. Optimization of the amounts of gD-RNA-synt to utilize as a template in the RT-qPCR assay. Real-time PCR analysis of cDNAs produced using three ten-fold dilutions (from 10^8 to 10^6 molecules diluited in RNase-water) of gD-RNA-synt as template and 10^{-2} U HIV-RT in RT reaction. (a) Amplification curves of one representative experiment where samples containing complete reaction mixtures (RT+, black text) and corresponding negative controls (RT-, red text) are depicted. Each RT- sample contained the same amount of RNA template used in RT+ samples. (b) Ct values of RT+ samples are reported as mean threshold cycle ± standard deviation from four replicates, while Ct values of RT- samples from four replicates are singularly reported. As shown, occasionally amplification of RT- sample corrisponding to 10^8 molecules of gD-RNA-synt template was detected at high Ct values.

Supplemental Table S1

Table S1. Comparison between the CT values obtained in the same experiment using total RNA or gD-RNA-synt as a template. Reaction conditions: 1X RT-Buffer, 0.2 mM dNTP mix, 0.5 µM gD-reverse primer, 0.025 U HIV-RT, 1 h at 37°C + 5 min at 90°C.

**RNA template**		
	**CT average**	***St.dev ±***
**Total RNA (150 ng)**	25.09	0.98
**gD-RNA-synt (10 ng)**	3.20	0.10

Supplemental Table S2

Table S2. Comparison between the CT values obtained in the same experiment using fixed amounts of total RNA or gD-RNA-synt as a template and variable amounts of HIV RT. Reaction conditions: 1X RT-Buffer, 0.2 mM dNTP mix, 0.5 µM gD-reverse primer, 1 h at 37°C + 5 min at 90°C.

RNA template	HIV-RT [U]	RT+		RT-		
		Average	*St.dev ±*	Ct_{v1}	Ct_{v2}	Ct_{v3}
Total RNA (150 ng)	2.5 x 10^{-2}	24.71	0.12	Undet	Undet	32.24
	2.5 x 10^{-3}	26.73	0.96	Undet	32.80	Undet
	2.5 x 10^{-4}	Undet	-	Undet	Undet	Undet
gD-RNA-synt (0.3 x 10^{-3} ng)	2.5 x 10^{-2}	22.40	0.18	Undet	Undet	Undet
	2.5 x 10^{-3}	24.26	0.04	Undet	Undet	Undet
	2.5 x 10^{-4}	30.63	0.50	Undet	Undet	Undet
